# Impact of gastrectomy on efficacy and safety of second‐line chemotherapy patients with advanced gastric cancer: Exploratory analysis of two randomized phase III trials

**DOI:** 10.1002/ags3.12880

**Published:** 2024-11-04

**Authors:** Kazuhiro Nishikawa, Wasaburo Koizumi, Akira Tsuburaya, Motoko Suzuki, Satoshi Morita, Kazumasa Fujitani, Yusuke Akamaru, Ken Shimada, Hisashi Hosaka, Ken Nishimura, Takaki Yoshikawa, Toshimasa Tsujinaka, Junichi Sakamoto

**Affiliations:** ^1^ Cancer Treatment Center OsakaInternational Medical & Science Center, Osaka Keisatsu Hospital Osaka Japan; ^2^ Kitasato University Sagamihara Kanagawa Japan; ^3^ Department of Surgery AOI Nanasawa Rehabilitation Hospital Atsugi Japan; ^4^ Department of Data Science National Cancer Center Hospital East Chiba Japan; ^5^ Department of Biomedical Statistics and Bioinformatics Kyoto University Graduate School of Medicine Kyoto Japan; ^6^ Department of Surgery Osaka General Medical Center Osaka Japan; ^7^ Department of Surgery Osaka Rosai Hospital Sakai City Osaka Japan; ^8^ Division of Medical Oncology Showa University Koto Totosu HospitalDepartment of Internal Medicine Tokyo Japan; ^9^ Department of Gastroenterology Gunma Prefectural Cancer Center Ohta Japan; ^10^ Department of Oncology Kitasato University Kitasato Institute Hospital Tokyo Japan; ^11^ Department of Gastric Surgery The National Hospital Organization National Cancer Center Hospital Tokyo Japan; ^12^ Izumi City General Hospital Izumi Osaka Japan; ^13^ Tokai Central Hospital Kakamigahara Japan

**Keywords:** advanced gastric cancer, impact of gastrectomy, irinotecan‐based chemotherapy, second‐line chemotherapy

## Abstract

**Aims:**

Second‐line chemotherapy (SLC) improves survival in advanced gastric cancer (AGC). Although many patients receiving SLC have undergone gastrectomy, the impact of gastrectomy on SLC remains unclear.

**Patients and Methods:**

The objective was to evaluate the impact of gastrectomy on SLC for AGC. A total of 290 eligible patients registered in two randomized phase III trials evaluating SLC for patients with AGC was classified into the prior gastrectomy group (PGG; *n* = 187) or the no gastrectomy group (NGG; *n* = 103). We compared overall survival (OS), progression‐free survival (PFS), overall response rate (ORR), disease control rate (DCR), and safety between these two groups. Adjusted OS and adjusted PFS were estimated using inverse probability of treatment weighting (IPTW).

**Results:**

The PGG had better performance status (*p* = 0.001), more prior platinum agent (*p* < 0.001), and more frequent peritoneal metastasis (*p* = 0.006) than the NGG. The PGG had significantly better OS (13.8 vs. 9.3 mo; hazard ratio [HR]: 0.59; *p* < 0.001) and PFS (4.7 vs. 2.8 mo; HR: 0.58; *p* < 0.001) than the NGG. The PGG had significantly better adjusted OS (13.8 vs. 10.0 mo; IPTW HR: 0.66; *p* = 0.01) and adjusted PFS (4.3 vs. 3.2 mo; IPTW HR: 0.71; *p* = 0.027) than the NGG. No significant differences were observed in ORR and DCR. The incidence of Grade 3 or worse adverse events did not differ between the two groups except for a high incidence of anemia and diarrhea in the NGG.

**Conclusion:**

Patients with previous gastrectomy are expected to have better survival outcomes when receiving second‐line irinotecan (IRI)‐based chemotherapy for AGC.

## Graphical abstract

In an analysis of two RCTs of IRI‐based second‐line chemotherapy for AGC, patients with prior gastrectomy had better OS and PFS than patients without prior gastrectomy. Furthermore, patients with prior gastrectomy had a better prognosis in OS and PFS adjusted for background factors as well.

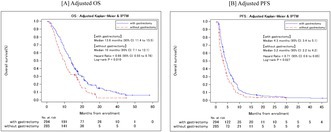

## 1 INTRODUCTION

Gastric cancer is one of the leading causes of cancer‐related deaths worldwide.^1^ Chemotherapy is the standard treatment for advanced gastric cancer (AGC), but in many cases second‐line chemotherapy (SLC) is also administered,^2^ as three trials demonstrated prolongation of survival with SLC using irinotecan (IRI) or docetaxel (DTX).^3^, ^4^, ^5^ IRI was used in several randomized trials as the SLC,^3^, ^4^, ^6^, ^7^, ^8^, ^9^, ^10^, ^11^ and IRI‐based chemotherapy is considered to be as good an option as SLC.

Chemotherapy is also given to patients who relapse after curative or palliative gastrectomy, but in addition to that in SLC, it is also given to patients who relapse early after adjuvant chemotherapy.^4^, ^7^, ^10^, ^11^ As a result, more than 30% of AGC patients undergo gastrectomy in clinical trials for SLC.^4^, ^7^, ^10^, ^11^, ^12^

Several clinical studies on first‐line chemotherapy for AGC^13^, ^14^ have reported that previous gastrectomy is an independent favorable prognostic factor, while a randomized trial, REGATTA,^15^ found no survival benefit for patients who underwent chemotherapy plus gastrectomy compared to those who received chemotherapy alone. A phase III trial comparing salvage chemotherapy with BSC reported that in a univariate analysis, prior surgery was not a significant predictor of overall survival (OS) compared with no prior surgery.^4^ However, no reports have examined the impact of gastrectomy on SLC in detail, and no randomized comparisons with or without gastrectomy have been reported in SLC. Thus, little is known about the effect of gastrectomy on SLC.

Two randomized phase III trials, the Tokyo Cooperative Oncology Group (TCOG) GI‐0801^10^ and the nonprofit organization Epidemiological & Clinical Research Information Network (ECRIN) TRICS,^11^ comparing two IRI‐based regimens, biweekly IRI plus cisplatin (CDDP) combination (BIRIP) and IRI monotherapy as SLC have been reported. Importantly, a total of more than 60% of patients who underwent gastrectomy were included in these two trials.

Therefore, in this exploratory analysis of two randomized phase III trials, to evaluate the impact of gastrectomy on SLC, we compared the safety and efficacy of IRI‐based chemotherapy in AGC patients who had undergone gastrectomy with those who had not.

## 2 PATIENTS AND METHODS

### 2.1 Study design

This study was an exploratory analysis using data of two randomized phase III trials, TCOG GI‐0801 and TRICS. Patients with previous gastrectomy were defined as the prior gastrectomy group (PGG), the remaining patients without prior gastrectomy were defined as the no gastrectomy group (NGG). We compared the efficacy and safety of IRI‐based SLC with PGG versus NGG.

Both the TCOG and the ECRIN groups gave their approval according to a formal protocol. We first verified the integrity of individual patient–level data (IPD) from the TCOG GI‐0801 trial^10^ and the TRICS trial^11^ following PRISMA‐IPD.^16^ All clinical data were extracted and held centrally at the data center of the ECRIN. The Institutional Review Boards or Ethics Committees of all participating centers reviewed and approved the protocol.

In both trials, patients were randomly allocated to BIRIP (CPT‐11, 60 mg/m^2^; CDDP, 30 mg/m^2^, q2w) or to IRI (150 mg/m^2^, q2w). In terms of patient enrollment, inclusion and exclusion criteria were reported previously.^10^, ^11^ Briefly, patients with histologically confirmed AGC were eligible who were refractory to the first‐line S‐1‐based chemotherapy (TCOG GI‐0801) or S‐1 monotherapy (TRICS), or that recurred during or within 6 mo after the completion of adjuvant therapy with S‐1. Other eligibility criteria were as follows: patients aged ≥20 y; an estimated life expectancy of at least 12 weeks; written informed consent; an Eastern Cooperative Oncology Group performance status (PS) of 2 or less (TCOG GI‐0801), 1 or less (TRICS); with adequate organ function (white blood cell count ≥4000/mm^3^ and ≤12 000 mm^3^, neutrophil count ≥2000/mm^3^, platelet count ≥100 000/mm^3^, hemoglobin level ≥8.0 g/dL, aspartate aminotransferase and alanine aminotransferase (ALT) levels ≤100 IU/L, total bilirubin level ≤1.50 mg/dL, and creatinine level ≤ upper limit of normal (TCOG GI‐0801), ≤1.20 mg/dL (TRICS)). Exclusion criteria included: history of antitumor therapy (except for S‐1‐based chemotherapy and surgery), additional malignancies, or significant comorbidities.

### 2.2 Assessment

Of the patients included in the two randomized phase III trials, only those who were eligible for each study were included in the efficacy and safety assessments. The efficacy endpoints of this study were OS, progression‐free survival (PFS), overall response rate (ORR), disease control rate (DCR), and adverse events (AEs), were compared between the groups with and without prior gastrectomy. Tumor responses were analyzed in patients with at least one measurable lesion at baseline using Response Evaluation Criteria in Solid Tumors (RECIST) version 1.0. Tumor responses were classified as complete response (CR), partial response (PR), stable disease (SD), or progressive disease (PD). The ORR was defined as the proportion of patients with CR or PR, and the DCR was defined as the proportion of patients with CR, PR, or SD. Grade 3 or worse adverse events were evaluated using the Common Terminology Criteria for Adverse Events (CTCAE) version 3.0.

### 2.3 Statistical methods

OS and PFS curves were constructed as time‐to‐event plots using the Kaplan–Meier method. Time‐to‐event curves were compared using the log‐rank test. HRs were estimated using Cox regression models. We used Fisher's exact test to compare ORRs between the two treatment groups. The confidence coefficient for the confidence interval for median OS, HR, and ORR was set to 95% (*p* < 0.05). Moreover, to adjust for confounding factors, we estimated OS and PFS using adjusted Kaplan–Meier curves with inverse probability weighting (IPTW). Adjusted HRs were estimated using a weighted Cox proportional hazards model with IPTW.^17^, ^18^ A logistic regression model with six covariates was used to estimate the propensity score: age, gender, Eastern Cooperative Oncology Group performance status (PS), histology, prior platinum agent, and measurable lesion. AE frequencies for Grade 3 or 4 were compared using Fisher's exact test. All clinical data were analyzed using SAS for Windows, v. 9.4 (SAS Institute, Cary, NC, USA).

## 3 RESULTS

### 3.1 Patient characteristics

**FIGURE 1:**
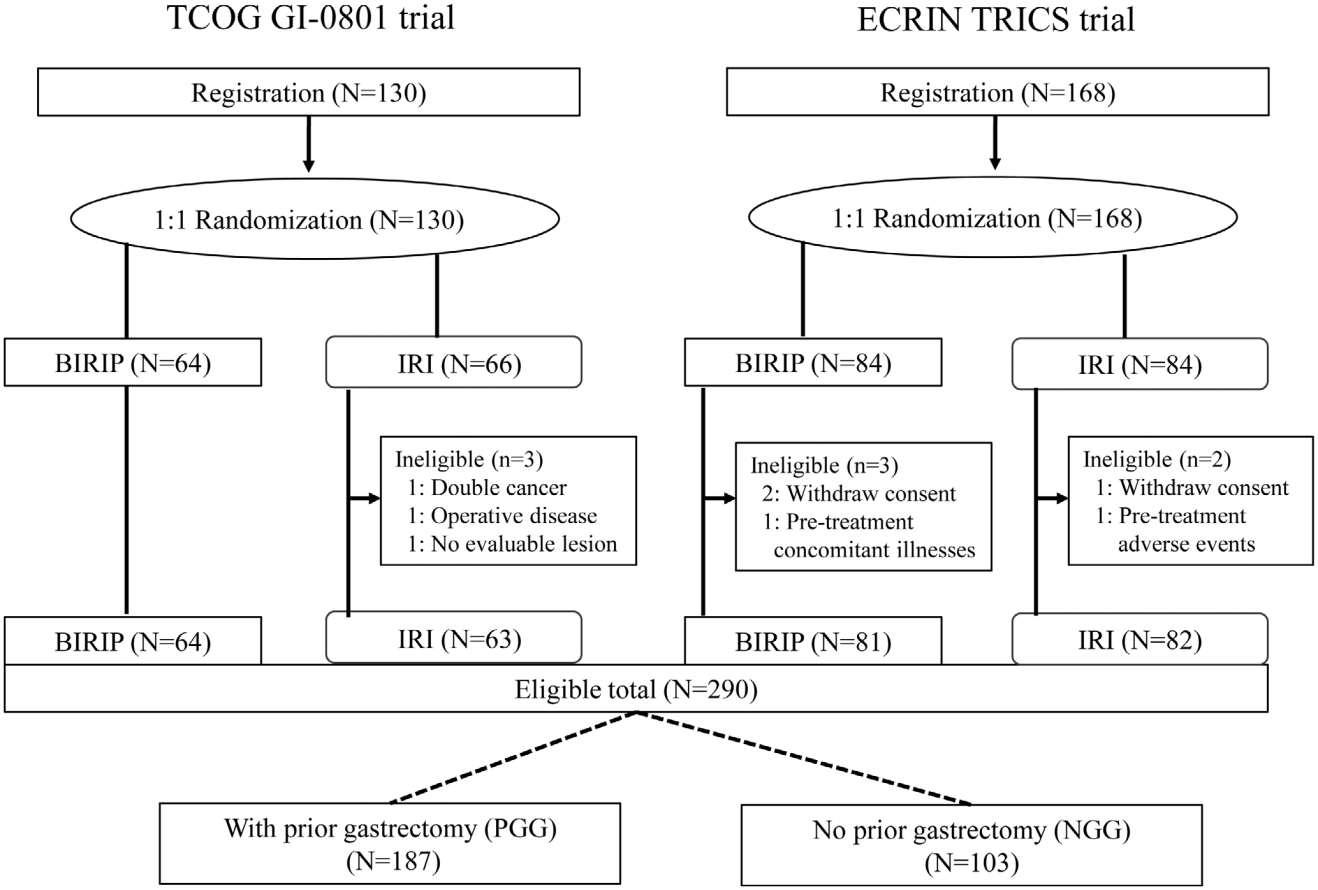
Patients’ disposition. BIRIP, biweekly irinotecan plus cisplatin; IRI, irinotecan; NGG, no gastrectomy group; PGG, prior gastrectomy group.

We extracted data of 127 patients eligible for the TCOG GI‐0801 trial (*N* = 130), and 163 patients eligible for the TRICS trial (*N* = 168) (Figure 1). The cumulative data of 290 eligible patients were evaluated. Previous gastrectomy was performed in 187 of 290 (64.5%) patients. PGG consisted of patients with tumor progression after first‐line S‐1‐based chemotherapy for advanced cancer with palliative resection, or recurrent cancer after curative resection, or patients who received S‐1‐based adjuvant chemotherapy with early relapse during or within 6 mo after the completion of adjuvant chemotherapy. The patient characteristics are shown in Table 1. PS was better in PGG than in NGG (*p* = 0.001). NGG (57 of 103 patients; 55.3%) had more prior platinum agent treatment than PGG (15 of 187 patients; 8.0%) (*p* < 0.001). A higher proportion of patients in NGG had peritoneal metastasis (22 of 103 patients; 21.4%) compared with PGG (17 of 187 patients; 9.1%) (*p* = 0.006). The remaining characteristics were generally similar between PGG and NGG.

**TABLE 1:** Patient characteristics according to prior gastrectomy.

Eligible patients (*n =* 290)	With prior gastrectomy (PGG)	Without prior gastrectomy (NGG)	*p*‐value
No. of patients	187 (64.5)	103 (35.5)	
Age			0.325
Median (range)	66 (35–87)	68 (29–87)	
Age			0.618
<65	77 (41.2)	39 (37.9)	
≥65	110 (58.8)	64 (62.1)	
Gender			0.226
Male	153 (81.8)	78 (75.7)	
Female	34 (18.2)	25 (24.3)	
ECOG PS			0.001
0	154 (82.4)	66 (64.1)	
1	33 (17.6)	37 (35.9)	
Histology			1.000
Intestinal	93 (49.7)	52 (50.5)	
Diffuse	94 (50.3)	51 (49.5)	
Regimen			0.326
IRI	98 (52.4)	47 (45.6)	
BIRIP	89 (47.6)	56 (54.4)	
Prior platinum agent			< 0.001
No	172 (92.0)	46 (44.7)	
Yes	15 (8.0)	57 (55.3)	
Measurable lesion			0.084
No	33 (17.6)	10 (9.7)	
Yes	154 (82.4)	93 (90.3)	
Peritoneal metastasis			0.006
No	170 (90.9)	81 (78.6)	
Yes	17 (9.1)	22 (21.4)	

Abbreviations: BIRIP, biweekly irinotecan plus cisplatin; ECOG, Eastern Cooperative Oncology Group; IRI, irinotecan; NGG, no gastrectomy group; PGG, prior gastrectomy group; PS, performance status.

### 3.2 Treatment

The median number of treatment courses was significantly higher in PGG (6 courses; range, 1–39) than in NGG (4 courses; range, 1–33) (*p* = 0.004) (Table 2). Treatment delays occurred more frequently in NGG than in PGG, but the difference was not significant (Table 2). Postprotocol therapy was administered 72.3% and 73.8% of patients in PGG and NGG, respectively (*p* = 0.784) (Table 2). The administered regimens consisted of mainly taxanes such as paclitaxel (PTX), DTX, and S‐1 plus DTX.

**TABLE 2:** Treatment according to prior gastrectomy.

Eligible patients (*n =* 290)	With prior gastrectomy (PGG)	Without prior gastrectomy (NGG)	*p*‐value
No. of patients	187 (64.5%)	103 (35.5%)	
Number of courses			0.004
Median (range)	6 (1–39)	4 (1–33)	
Treatment delays			0.052
Yes	115 (61.5%)	75 (72.8%)	
No	72 (38.5%)	28 (27.2%)	
Postprotocol therapy			0.784
Yes	133 (72.3%)	76 (73.8%)	
No	51 (27.7%)	27 (26.2%)	
Unknown	3	0	
Postprotocol regimen			
PTX	91 (49.5%)	49 (47.6%)	
DTX	19 (10.3%)	13 (12.6%)	
S‐1/DTX	16 (8.7%)	3 (2.9%)	
IRI	15 (8.2%)	6 (5.8%)	
BIRIP	11 (6.0%)	4 (3.9%)	
Other	20 (10.9%)	3 (2.9%)	
Unknown	3	0	

Abbreviations: BIRIP, biweekly irinotecan plus cisplatin; DTX, docetaxel; IRI, irinotecan; NGG, no gastrectomy group; PGG, prior gastrectomy group; PTX, paclitaxel.

### 3.3 Survival

**FIGURE 2:**
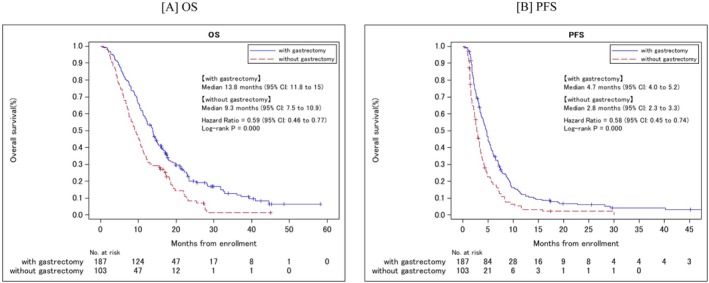
Kaplan–Meier curves for OS (A) and PFS (B) by prior gastrectomy. OS, overall survival; PFS, progression‐free survival.

The OS was significantly longer in PGG (13.8 mo [95% CI: 11.8–15.0]) than in NGG (9.3 mo [95% CI: 7.5–10.9; HR 0.59; 95% CI: 0.46–0.77, *p* < 0.001]) (Figure 2A). Furthermore, PGG had significantly better PFS than NGG (4.7 [95% CI: 4.0–5.2] vs. 2.8 [95% CI: 2.3–3.3]; HR: 0.58; 95% CI: 0.45–0.74; *p* < 0.001); (Figure 2B).

**FIGURE 3:**
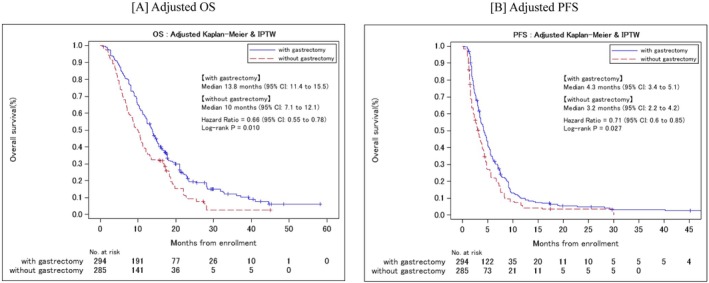
Adjusted Kaplan–Meier curves for adjusted OS (A) and adjusted PFS (B) with inverse probability weight by prior gastrectomy. IPTW, inverse probability of treatment weighting; OS, overall survival; PFS, progression‐free survival.

The adjusted median OS was 13.8 mo (95% CI:11.4–15.5) in PGG and 10.0 mo (95% CI:7.1–12.1) in NGG (IPTW HR: 0.66; 95% CI:0.55–0.78; *p* = 0.01; Figure 3A). The adjusted median PFS was 4.3 mo (95% CI:3.4–5.1) in PGG and 3.2 mo (95% CI:2.2–4.2) in NGG (IPTW HR: 0.71; 95% CI:0.6–0.85; *p* = 0.027); (Figure 3B). Thus, PGG had significantly better‐adjusted OS and PFS than NGG.

### 3.4 Tumor responses

Of the 154 patients in PGG who had measurable disease, 4 achieved CR, 26 achieved PR, and 74 achieved SD. Of the 93 patients in NGG who had measurable disease, 15 achieved PR, and 43 achieved SD. The ORR was 19.5% [95% CI: 13.2–25.7] in PGG and 16.1% [95% CI: 8.7–23.6] in NGG, indicating no significant difference (*p* = 0.610) (Table 3). And the DCR was 67.5% [95% CI: 60.1–74.9] in PGG and 62.4% [95% CI: 52.5–72.2] in NGG, indicating no significant difference (*p* = 0.411) (Table 3).

**TABLE 3:** Clinical responses according to prior gastrectomy.

	With prior gastrectomy (PGG)	Without prior gastrectomy (NGG)	*p*‐value
No. of patients	154	93	
Best overall response (%)			
CR	4 (2.6)	0 (0.0)	
PR	26 (16.9)	15 (16.1)	
SD	74 (48.1)	43 (46.2)	
PD	38 (24.7)	34 (36.6)	
NE	12 (7.8)	1 (1.1)	
ORR (CR, PR)	30 (19.5)	15 (16.1)	0.610
95% CI	(13.2–25.7)	(8.7–23.6)	
DCR (CR, PR, SD)	104 (67.5)	58 (62.4)	0.411
95% CI	(60.1–74.9)	(52.5–72.2)	

Abbreviations: CI, confidence interval; CR, complete response; DCR, disease control rate; PGG, prior gastrectomy group; NE, not evaluable; NGG, no gastrectomy group; ORR, objective response rate; PD, progressive disease; PR, partial response; SD, stable disease.

### 3.5 Adverse events

The proportions of patients with Grade 3 or worse AEs are shown in Table 4. The incidence of anemia (≥Grade 3) and diarrhea (≥Grade 3) were higher in NGG (19.4%, 5.8%) than PGG (10.2%, 1.1%). The incidence of other Grade 3 or worse AEs did not differ between the two groups (Table 4).

**TABLE 4:** Grade 3 or greater adverse events according to prior gastrectomy.

Adverse events	With prior gastrectomy (PGG), (*n =* 187)	Without prior gastrectomy (NGG), (*n =* 103)	*p*‐value
≥G3	*n* (%)	*n* (%)	
Leukopenia	24 (12.8)	17 (16.5)	0.385
Neutropenia	63 (33.7)	36 (35.0)	0.897
Anemia	19 (10.2)	20 (19.4)	0.032
Thrombocytopenia	1 (0.5)	1 (1.0)	1.000
Febrile neutropenia	1 (0.5)	2 (1.9)	0.288
AST increased	10 (5.3)	2 (1.9)	0.224
ALT increased	6 (3.2)	3 (2.9)	1.000
T‐Bil increased	4 (2.1)	2 (1.9)	1.000
ALP increased	6 (3.2)	6 (5.8)	0.357
Creatinine increased	1 (0.5)	1 (1.0)	1.000
Hyperkalemia	2 (1.1)	2 (1.9)	0.617
Hyponatremia	1 (0.5)	2 (1.9)	0.288
Fatigue	1 (0.5)	0 (0.0)	1.000
Anorexia	13 (7.0)	11 (10.7)	0.274
Nausea	7 (3.7)	6 (5.8)	0.554
Vomiting	2 (1.1)	2 (1.9)	0.617
Diarrhea	2 (1.1)	6 (5.8)	0.026

Abbreviations: ALP, alkaline phosphatase; ALT, alanine aminotransferase; AST, aspartate aminotransferase; NGG, no gastrectomy group; PGG, prior gastrectomy group; T‐Bil, total bilirubin.

## 4 DISCUSSION

Our exploratory study of the two randomized trials showed that PGG demonstrated significantly longer OS (HR 0.59) and PFS (HR 0.58) than NGG, as well as adjusted OS (IPTW HR: 0.66) and adjusted PFS (IPTW HR: 0.71). The incidence of Grade 3 or worse AEs did not differ between the two groups except for a high incidence of anemia and diarrhea in NGG. To our knowledge, this analysis is the first to show that patients who have undergone gastrectomy have superior survival and safety compared to those who have not undergone gastrectomy in SLC for AGC.

Because our analysis was not preplanned, baseline patient characteristics were not well balanced between the two groups. Patients undergoing surgical resection need to be able to tolerate surgery; thus, it is conceivable that those patients were younger and had better PS. In addition, prior platinum agent therapy and peritoneal metastases, which were more frequent in the NGG, are likely to influence survival outcomes. For that reason, we evaluated adjusted OS and adjusted PFS, which were constructed with adjusted Kaplan–Meier curves with inverse probability weighting, to adjust for confounding factors.^17^, ^18^ The superiority of PGG over NGG in OS and PFS not only in univariate analysis but also in IPTW analysis seems to confirm the fact that patients with prior gastrectomy have a better prognosis than those without gastrectomy.

In patients receiving chemotherapy, gastrectomy can have both a negative and positive impact on survival. When chemotherapy is administered immediately after gastrectomy, the negative impact of gastrectomy has been reported to include delayed chemotherapy initiation due to invasive surgery, increased toxicity of chemotherapy, and decreased chemotherapy tolerance.^15^, ^19^ Such effects are associated with tumor growth due to immunosuppression and may have adverse effects in the form of nutritional disturbances with relatively long‐term consequences, especially after total gastrectomy.^15^, ^20^, ^21^ However, in our study as the SLC setting, the incidence of anemia (>Grade 3) and diarrhea (>Grade 3) was rather the opposite, with NGG (19.4% and 5.8%) having higher rates than PGG (10.2% and 1.1%). We do not know why diarrhea was more common in NGG, but as for anemia, it may be that anemia was less common in PGG due to the fact that the primary lesion, often a potential source of bleeding, was removed. Furthermore, treatment delays tended to be more common in NGG, rather than the other way around. Because of SLC, our cases might be due to having enough period to recover from the negative impact of gastrectomy.

By contrast, the positive impact of gastrectomy may be a reduction in tumor volume. Patients with prior gastrectomy might be in a state of reduced magnitude of potentially immunosuppressive tumor burden and less new source of metastasis. It is therefore possible that durable systemic chemotherapy is being promoted. In first‐line chemotherapy, findings from several clinical studies have shown that past gastrectomy along with a small number of metastatic sites are independent favorable prognostic factors, which suggest the relevance of reducing tumor burden for achieving longer OS.^13^, ^14^ On the other hand, there are a few clinical trial reports on the effect of gastrectomy on survival in second‐ or later‐line chemotherapy. Kang JH reported that prior surgery was not the significant predictor of OS in their univariate analysis compared with no prior surgery (HR 0.862; *p* = 0.736) in their phase III trial comparing salvage chemotherapy with BSC alone.^6^ As the third‐ or later‐line setting, in the phase 3 ATTRACTION‐2 trial,^22^ nivolumab improved OS compared with placebo in subgroups of patients who had undergone gastrectomy (HR, 0.61) or who had not (HR, 0.69). In the phase 3 TAGS trial,^23^ trifluridine/tipiracil improved OS compared with placebo in subgroups of patients who had undergone gastrectomy (HR, 0.57) or had not (HR, 0.80). In subgroup analyses of both trials, patients who had not undergone gastrectomy also had improved OS after receiving treatment with nivolumab and FTD/TPI, respectively, but this benefit was more pronounced in the gastrectomy subgroup. Our study also showed a similar survival benefit, and the course of treatment was significantly higher for PGG. The most likely reason for the better survival of PGG compared to NGG in our analysis is the positive impact of gastrectomy in terms of tumor volume reduction. In other words, the reason for the difference between our study and the randomized REGATTA trial could be that the positive impact of gastrectomy in terms of tumor volume reduction remained the same for both, but the negative impact of gastrectomy decreased over time because our study was in the second‐line treatment setting.

The ratio of the prior platinum agent was 8% of PGG, while it was 55.3% of NGG in the background factors, which is considered to be one of the reasons for this result of univariate analysis before adjustment. It is generally assumed that the prognosis is poor after the use of multiple drugs in pretreatment; moreover, the strategy is considered negative because continuous use of the drug that caused progression in the previous therapy does not improve survival outcomes.^8^, ^24^, ^25^ This cause for the poor survival of the NGG group in the univariate analysis may have been that about 30% (BIRIP 54.4%, Prior platinum 55.3%) of patients in the NGG had to continue on platinum drugs. Another reason for a better survival result of univariate analysis before adjusted in PGG is that the percentage of peritoneal metastases was higher in NGG (21.4%) than in PGG (9.1%). In general, peritoneal metastases with massive ascites and cancer cachexia are very difficult to treat even with chemotherapy and have a poor prognosis,^26^ but the efficacy of chemotherapy and the prognosis of patients with peritoneal metastases has been reported to depend on the severity of peritoneal metastases.^27^ Moreover, in the two randomized trials on which this analysis was based, such severe cases with peritoneal metastases fell outside the eligibility criteria.^10^, ^11^

There are several limitations to this study. This exploratory analysis was not planned before the two randomized phase III trials were ongoing; thus, the results should be interpreted with caution. And this study only included Japanese patients and ethnic differences between Asian and Western patients could have affected the overall results. Furthermore, application of the present results may be limited, as combination therapies containing platinum, such as CAPOX and SOX, are currently among the standard adjuvant treatments,^28^, ^29^ and the standard of care for HER2‐negative AGC is an anti–programmed death receptor‐1 antibody with oxaliplatin and fluoropyrimidine.^30^, ^31^ Lastly, while the two randomized phase III trials were ongoing, the RAINBOW trial^12^ reported that ramucirumab plus paclitaxel combination therapy is currently thought to be the standard SLC, and IRI is considered as third‐ or later‐line. The negative effects of increased chemotherapy AEs due to gastrectomy and decreased chemotherapy intensity due to intolerance to chemotherapy due to gastrectomy are expected to be similar or even less pronounced in the third‐line and subsequent lines of treatment. Therefore, it can be inferred that the results obtained in this study for the SLC are also applicable to the third‐line chemotherapy and beyond.

In conclusion, although the mechanism causing the difference in efficacy between PGG and NGG cannot be completely explained biologically, IRI‐based chemotherapy may be a more effective and more clinically appropriate option for patients with prior gastrectomy in SLC.

## AUTHOR CONTRIBUTIONS

**Kazuhiro Nishikawa:** Conceptualization; data curation; investigation; methodology; writing – original draft. **Wasaburo Koizumi:** Conceptualization; data curation; methodology; writing – review and editing. **Akira Tsuburaya:** Conceptualization; project administration; writing – review and editing. **Motoko Suzuki:** Formal analysis; methodology; writing – review and editing. **Satoshi Morita:** Investigation; validation; writing – review and editing. **Kazumasa Fujitani:** Data curation; methodology; writing – review and editing. **Yusuke Akamaru:** Data curation; writing – review and editing. **Ken Shimada:** Data curation; writing – review and editing. **Hisashi Hosaka:** Data curation; writing – review and editing. **Ken Nishimura:** Data curation; writing – review and editing. **Takaki Yoshikawa:** Data curation; investigation; methodology; validation; writing – review and editing. **Toshimasa Tsujinaka:** Methodology; supervision; writing – review and editing. **Junichi Sakamoto:** Funding acquisition; resources; supervision; writing – review and editing.

## FUNDING INFORMATION

This work was supported, in part, by the nonprofit organization Epidemiological and Clinical Research Information Network (ECRIN) (no grant numbers apply).

## CONFLICT OF INTEREST STATEMENT

Satoshi Morita has received honoraria for lectures from AstraZeneca K. K., Bristol‐Myers Squibb Co. Ltd., Chugai Pharmaceutical Co. Ltd., Eli Lilly Japan K.K., MSD K.K., and Ono Pharmaceutical Co. Ltd. All remaining authors declare no conflicts of interest.

## ETHICS STATEMENT

Approval of the research protocol by an Institutional Reviewer Board: This trial was conducted in compliance with the ethical principles of the Declaration of Helsinki and the Ethical Guidelines for Clinical Studies of the Japanese Ministry of Health, Labour and Welfare. This trial was approved by the Institutional Review Boards or Ethics Committees at all participating centers.

Informed Consent: N/a.

Registry and the Registration No. of the study/trial: This trial was registered with Clinical Trials.gov (UMIN 000025367).

Animal Studies: N/a.

## CLINICAL TRIAL REGISTRATION

UMIN 000025367.
